# Howling Detection and Suppression Based on Segmented Notch Filtering

**DOI:** 10.3390/s21238062

**Published:** 2021-12-02

**Authors:** Yanping Li, Xiangdong Huang, Yi Zheng, Zhongke Gao, Lei Kou, Junhe Wan

**Affiliations:** 1 School of Electrical and Information Engineering, Tianjin University, Tianjin 300072, China; 18678989360@126.com (Y.L.); xdhuang@tju.edu.cn (X.H.); zhongkegao@tju.edu.cn (Z.G.); 2 Institute of Oceanographic Instrumentation, Qilu University of Technology (Shandong Academy of Sciences), 37 Miaoling Road, Qingdao 266000, China; zhengy@qlu.edu.cn (Y.Z.); koulei1991@hotmail.com (L.K.)

**Keywords:** center frequency control, FIR notch filter, howling detection, howling suppression

## Abstract

The existing adaptive echo cancellation based howling (typically in hearing aids) removal methods have several drawbacks such as insufficient attenuation of the howling component, slow response and nonlinear distortion. To solve these problems, we propose a segmented notch filtering based scheme. Specifically, firstly, it is proved that the attenuation value can reach −330 dB at any detected howling frequency; secondly, the filter coefficients can be readily calculated by a closed-form formula, yielding a fast response to the sudden howling accident; thirdly, the closed-form formula of this filter is theoretically an even function, indicating that this filter possesses a linear transfer characteristic. In combination with proper segmentation and precisely removing these transient samples arising from FIR (Finite Impulsive Response) filtering, nonlinear distortion can be entirely avoided. Experimental results show that our proposed scheme can not only accurately estimate the howling frequency, but can also completely remove it, which yields a high-quality output waveform with a recovery SNR of about 22 dB. Therefore, the proposed segmented notching based scheme possesses vast potential for hearing aid development and other relevant applications.

## 1. Introduction

Hearing, which acts as a crucial link of communication with the surroundings, is one of the most important senses of human beings. In recent years, global noise pollution and the aging of the world population have led to an increasing number of people with hearing loss [1]. Scientists are constantly adopting various methods to help hearing-impaired patients to improve hearing. Wearing hearing aids is one of the most commonly used methods to compensate patients for hearing loss. A hearing aid is mainly composed of microphone, amplifier, speaker, battery, various volume and tone control knobs and other components. The development of hearing aids has experienced the following five periods: (1) the original set of sound hearing aid period; (2) carbon element hearing aid period; (3) vacuum tube hearing aid period; (4) transistor and integrated circuit hearing aid period; and (5) digital hearing aid period [2]. Nowadays, the digital hearing aid is mainstream [3,4].

In a digital hearing aid, sound collected in the microphone is converted into an amplified electrical signal, and then applied with a compensation operation which helps to improve the hearing of wearers. However, a digital hearing aid has a thorny problem—howling [5]. This defect arises from the following facts: a hearing aid is actually realized by applying distinct dynamic gains across different frequency bands to the input speech. However, the existence of the feedback path from the speaker to the microphone (since they have to be put closely in an ear) occasionally converts these unequal gains into a positive feedback, and thus the howling, which behaves as high-intensity vibration at a certain frequency and thus makes the wearer uncomfortable. This greatly degrades the performance of a hearing aid. Hence, some compensation operations need to be developed to suppress this howling.

There are three main methods of howling suppression: gain attenuation [6], adaptive echo cancellation [7,8,9,10], and notch filtering [11,12].

Gain attenuation is realized by simply reducing all the preset gains in the echo channel according to the amplitude of the howling [13]. This method has the advantage of low power consumption, but it suffers from the quality loss of the expected frequency components.

The adaptive echo cancellation method essentially utilizes the principle that the howling is incurred by the unexpected feedback from the speaker to the microphone. Hence, it builds up an adaptive filter, whose output is subtracted from the speaker signal as the final sound fed to the wearer. As theoretically proved in Section 2.2, if this adaptive filter exactly converges to the impulse response of the echo feedback channel, the howling will be completely cancelled. However, since the howling occurs suddenly and sharply with a large amplitude, it is actually very difficult to realize this convergence. Specifically, the tracking performance of this adaptive filter is always far from the expectation, which requires a proper selection of the adaptive criteria and the precise setting of the adaptive step size [14].

To overcome the limitations of the adaptive echo cancellation method and achieve a better howling cancellation, many researchers incorporated the notch filtering technique into the adaptive filtering scheme. For example, Liang [15] combines the variable step normalized least mean square(VNLMS) algorithm with the notch filter algorithm to quickly suppress the howling. A. Sogami [16] proposed a howling canceller, which cancels howling by using a cascade notch filter designed from a distance between a loudspeaker and a microphone. It adaptively adjusts the nulls to eliminate howling and to keep speech components. A. Pandey [17] presents a new method that switches to a least-squares adaptation scheme with linear complexity at the onset of howling. It reformulates the least-squares estimate into a Toeplitz system and is solved with a direct Toeplitz solver. With the notch filtering technique integrated, these works achieve a better howling suppression effect than the sole adaptive echo cancellation method does.

From the viewpoint of frequency domain analysis, as [13] pointed out, the spectrum of the howling exhibits a large peak at a particular frequency point. This indicates that, as long as the transfer characteristic of the notch filter is sufficiently ideal, the howling can be completely removed by the notch filter without the help of the adaptive filter. In our opinion, a notch filter design suitable for howling removal should possess an arbitrary realizable notching frequency point, a sufficiently flat passband, a linear phase characteristic and low design complexity. Nevertheless, the existing notch filter design (such as the Parks–McClellan technique [18], the maximally flat (MF) method [19] and the equiripple (ER) method proposed by Zahradnik [20]) does not satisfy these requirements. Therefore, the core problem of howling suppression lies in the design of a high-performance notch filter. The major contributions of this paper are listed as follows:

1. On the basis of our previous work in [21], this paper proposes a closed-form FIR notch filter design, concurrently satisfying the above four conditions (i.e., arbitrary realizable notching frequency point, sufficiently flat passband, linear phase characteristic and low design complexity);
2. Further, to ensure that howling suppression can work continuously while remaining the linear phase characteristic, we design a feasible segmentation based scheme consisting of an energy detector, spectrum corrector and a segmented notch filter;
3. This paper actually develops a high-efficiency howling suppression mode, which can not only shorten the howling detection to an extent that a wearer can hardly recognize this accident, but also readily removes the howling without incurring any phase distortions.

The rest of this paper is organized as follows. In Section 2, we introduce the reasons for howling generation and the problems of the echo cancellation based removal method. In Section 3, we propose a new hearing aid design scheme with a howling detection and notch filter. Section 4 introduces the design of closed-form notch filter in detail, which includes the general convolution window of an FIR filter, derivation of the closed-form formula, and extra data extensions. Section 5 shows numerical results from the aspects of comparison between the proposed notch filter and the conventional IIR notch filter, the demonstration of the proposed closed-form notch filter and the linear-phase characteristic, different howling frequencies, different filter orders, and different data extension lengths. In Section 6, we draw a conclusion that the proposed scheme is of high efficiency and low computing complexity, which has vast potential for future development.

## 2. Howling Generation and the Echo Cancellation Based Removal Method

### 2.1. Howling Generation

**Figure 1:**
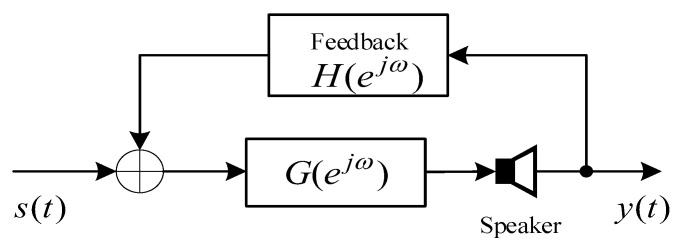
The model diagram of the hearing aid system.

Figure 1 shows the block diagram of the hearing aid model, where s(t) represents the input speech, y(t) represents the output signal, $G(e^{j\omega })$ represents the pre-set system function for the sake of hearing compensation and noise reduction, and $H(e^{j\omega })$ represents the real echo feedback path from the loudspeaker to the microphone.

On the basis of the model illustrated in Figure 1, one can derive the transfer function Q(z) from input to speaker as:

(1) $$Q\left(z\right)=\frac{Y(z)}{S(z)}=\frac{G(z)}{1-H(z)G(z)}.$$

It can be inferred from (Equation (1)) that when both of the following two conditions are satisfied,

(2) $$G\left(e^{j\omega }\right)H\left(e^{j\omega }\right)\to 1,$$

(3) $$∠G\left(e^{j\omega }\right)H\left(e^{j\omega }\right)=n\cdot 360\;n=1,2,\ldots ,$$

then the denominator in (Equation (1)) gets close to 0, and thus the total transfer function Q(z) tends to be extremely large, which inevitably gives rise to the occurrence of howling or whistling. Once howling happens, there surely exists a pole point of Q(z) such that 1−H(z)G(z)→0. Moreover, as is well known, each pole point corresponds to a special frequency component, resulting in the waveform of a howling appearing like a single-frequency sinusoid oscillation.

Practically, howling is closely related to the term H(z) in the denominator of Equation (1), which is occasionally affected by a wearer’s inadvertent actions [22] (such as slightly touching the edge of the loudspeaker or microphone). When this occurrence leads to the satisfaction of the condition Equations (2) and (3), howling happens.

Moreover, the unpredictable occurrence of howling raises several requirements as follows: (1) Howling detection should be conducted persistently, which actually requires that the detection algorithm consumes low computational complexity; (2) the howling suppression algorithm should possess a fast response to this occurrence; (3) the howling suppression algorithm should completely remove the howling component and should not incur any phase distortion arising from this occurrence.

### 2.2. Problems of the Echo Cancellation Based Removal Method

**Figure 2:**
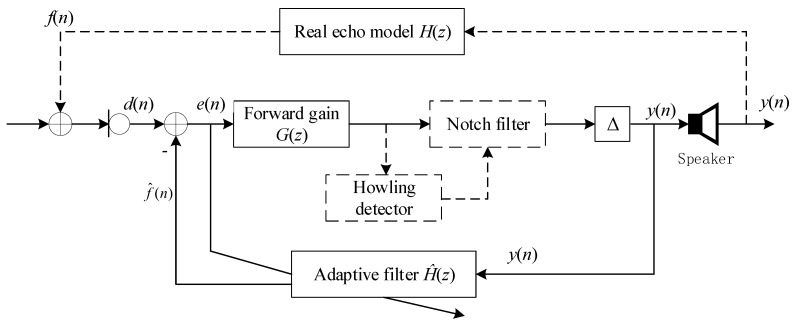
Echo cancellation algorithm model with adaptive filter for hearing aids.

To compensate for the loss arising from the occasional howling, one mainstream approach is to add an adaptive filter based loop inside the hearing aid (proposed by [15]), as illustrated in Figure 2.

Let $\hat{H}\left(z\right)$ be the transfer function of the adaptive filter. With this feedback loop added, one can derive the whole transfer function Q(z) of the hearing aid as:

(4) $$Q\left(z\right)=\frac{Y(z)}{S(z)}=\frac{G(z)}{1-[H\left(z\right)-\hat{H}\left(z\right)]G\left(z\right)}.$$

However, practically, this adaptive filter based echo cancellation scheme can hardly work well. The reasons are as follows:

1. Since the echo path H(z) behaves as significant randomness, its impulse response length can also hardly be predicted, resulting in the adaptive filter $\hat{H}\left(z\right)$ being unable to be specified with a fixed length (i.e., the delay amounts in Figure 2 can hardly be properly specified).
2. As is well known, any adaptive filter cannot overcome the trade-off between fast convergence and small stable-state residual error. If fast convergence is preferred (with a large adaptive step size specified), large stable-state residual error inevitably exists, resulting in the wearer still feeling the small-level howling after convergence. Nevertheless, if a small stable-state residual error is preferred (with a small adaptive step size specified), this adaptive filter has to experience a long-duration transition process, resulting in the wearer having to also tolerate a long-duration howling.
3. Another drawback lies in: as Figure 2 depicted, if $\hat{H}\left(z\right)$ does not approximate H(z) well, the transfer function Q(z) is equivalent to an infinite impulsive response system, which potentially hides a risk of instability, once any pole of the denominator of Q(z) falls outside the unit circle.

Since the adaptive filter cannot eliminate the howling solely, one improvement measure is to cascade the forward gain G(z) with a notch filter (as dotted line plots). Nevertheless, it is still intractable to well match the adaptive filter and the notch filter, since both of them require that a series of parameters be properly set.

## 3. The Proposed Scheme

### 3.1. Block Diagram

**Figure 3:**
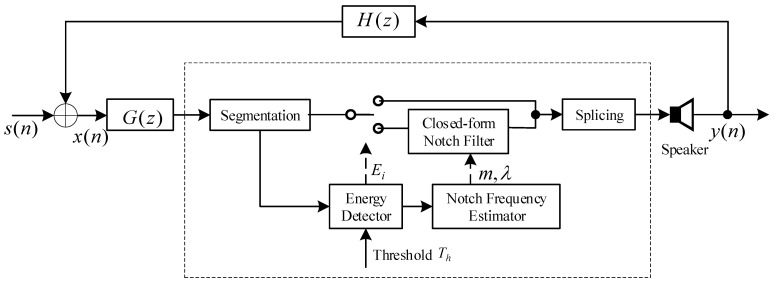
The hearing aid model diagram with howling detection and a notch filter.

Figure 3 shows our proposed hearing aid model diagram with howling detection and a notch filter. Different from the illustration in Figure 2, our proposed scheme completely leaves out the adaptive filter, which greatly reduces the system cost and improves the operation efficiency. Instead, we added a segmenting and splicing pair. In particular, the core part of this scheme lies in the closed-form notch filter, which is triggered by an energy detector and a frequency estimator.

To emphasize, as Figure 3 illustrated, the proposed dehowling module is just placed before the speaker, indicating that the proposed technique is no more than a post-processing measure of removing the howling effect rather than entirely removing all the distortions arising from the feedback channel. Actually, the existence of a feedback channel brings both an annoying large-amplitude howling effect and other distortions. In particular, as a post-processing measure, the proposed technique only aims to remove the howling effect and allows reserving of the other distortions. Practically, their amplitudes are much smaller than the howlings. As a result, once the conspicuous howling effect is removed completely, the remaining other small distortions are still acceptable for the hearing aid wearers.

### 3.2. Individual Noncore Parts

#### 3.2.1. Segmenting and Splicing

Denote x(n) as the output of the forward gain G(z) and specify the subsegment size as *P*. Therefore, the input data are subdivided into a series of subsegments: $x_{0}$, $x_{1}$, …, $x_{i}$, …, among which the *i*-th subsegment $x_{i}=\left[x_{i}\left(0\right)\;x_{i}\left(1\right),\cdots ,x_{i}\left(P-1\right)\right]=\left[x\left(iP\right)\;x\left(iP+1\right),\cdots ,x\left(iP+P-1\right)\right]$. As Figure 3 depicted, $x_{i}$ experiences the dehowling processing by the energy detector, the frequency estimator and the notch filter, and thus another subsegment $y_{i}=\left[y_{i}\left(0\right)\;y_{i}\left(1\right),\cdots ,y_{i}\left(P-1\right)\right]$ is outputted. Further, just splicing all the subsegments $y_{i},\;i=0,1,\cdots ,$ yields the final samples y(n) fed to the loudspeaker.

#### 3.2.2. Energy Detector

As mentioned above, since the howling leads to the consequence that the denominator of Q(z) approximates 0, that is, 1−H(z)G(z)→0, its waveform tends to be with a large amplitude, indicating that the occurrence of howling allows detection by a threshold $T_{h}$. Specifically, for the *i*-th subsegment, if its energy $E_{i}>T_{h}$, it is deemed that howling happens. Otherwise, no howling occurs.

Since energy detection needs to be conducted at all times, that is, whether howling occurs or not, this detector must be of very low computational cost. Clearly, this energy detector only involves a simple square summation operation and thus meets this requirement.

#### 3.2.3. Notch Frequency Estimator

Once howling occurrence is detected, it is further needed to estimate the howling frequency, which is commonly realized by directly implementing Discrete Fourier Transform (DFT) on the segmentation and searching out the spectral peak. Nevertheless, limited by DFT’s inherent pick fence effect, this estimator can only yield a rough result. To achieve a high accuracy, we resort to the ratio-based hamming-windowed spectrum corrector [23,24], which consists of the following three steps (assume that the sampling rate is $f_{s}$ and the segment size is *P*).

- **Step 1** Implement hamming-windowed DFT on the input data segment x to acquire the DFT spectra X(0),⋯,X(P−1), among which the peak spectral position is recorded as *m*.
- **Step 2** Calculate the amplitude ratio *v* between X(m) and its subpeak neighbor, that is,
  (5) $$v=\frac{\left|X(m)\right|}{\max\left|X(m-1)|,|X(m+1)\right|}.$$
- **Step 3** Calculate a fractional frequency offset λ as
  (6) $$\;\;\;\;\;\;\;\;\lambda =\left\{\begin{array}{cc} \frac{2-v}{1+v},\;\; & \mathrm{if}|X(m+1)|>|X(m-1)|\;\;\;\;\\ -\frac{2-v}{1+v},\;\; & \mathrm{else} \end{array}\right.$$

Accordingly, the frequency estimate is ${\hat{f}}_{0}=\left(m+\lambda \right)f_{s}/P$.

To emphasize, both the integer peak index *m* and the fractional frequency offset λ need to be fed into the core part—the design of the closed-form notch filter.

## 4. Design of the Closed-Form Notch Filter

### 4.1. Requirements of the Notch Filter Design

In practice, as the core part of our proposed scheme, the notch filter design should meet the following requirements:

1. To ensure that the hearing aid works efficiently when howling occurs, the notch filter design should be of low complexity;
2. To ensure that any howling event is properly dealt with, the notching frequency should be realized at an arbitrary frequency position;
3. To ensure that the frequency component of howling is significantly removed, the notch filter should possess large attenuation at the notch frequency;
4. To ensure that no speech distortion occurs (especially near the edge of adjacent segments), the notch filter should be of linear-phase characteristic.

It should be emphasized that the above four requirements cannot be satisfied by the existing notch filter designs [25,26]. Hence, we proposed a closed-form notch filter design, which is derived on the basis of our previous work—the convolution window based FIR filter design [21].

### 4.2. General Convolution Window Based FIR Filter Design

As [21] pointed out, given an *N*-length frequency vector H satisfying

(7) H(k)=H(N−k),k=1,2,⋯,N−1,

then, one can readily acquire a (2N−1) linear-phase FIR filter g=[g(−N+1),⋯,g(0),⋯,g(N−1)] in terms of the following three-step procedure.

- **Step 1** Implement IDFT on a specified frequency vector H to acquire h=[h(0),h(1),⋯, h(N−1)]. Then, insert the replica of h(1)∼h(N−1) into the left part of h to obtain the extended vector $h^{\prime }$.
- **Step 2** Implement normalized convolution between a commonly-used *N*-length window f(n) and an *N*-length rectangular window to obtain the (2N−1)-length window $w_{c}=\left[w_{c}\left(-N+1\right),\cdots ,w_{c}\left(0\right),\cdots ,w_{c}\left(N-1\right)\right]$.
- **Step 3** Implement the product on the corresponding elements of $h^{\prime }$ and $w_{c}$ to generate the final filter g(n),−N+1≤n≤N−1.

Theoretically, the filter coefficient g(n) equals [21]

(8) $$g\left(n\right)=h\left(n\right)w_{c}\left(n\right),\;-N+1\leq n\leq N-1,$$

where $w_{c}\left(n\right)$ refers to the convolution window generated by convolving an *N*-length symmetric window f and the reversed rectangular window $R_{N}$, that is,

(9) $$w_{c}\left(n\right)=f\left(n\right)*R_{N}\left(-n\right),n=-N+1,\cdots ,N-1,$$

where $w_{c}\left(n\right)$ needs to be normalized by a factor *C* defined as:

(10) $$C=\sum_{n=0}^{N-1}f(n).$$

The term h(n) in Equation (8) is the extended IDFT of H(k) defined as:

(11) $$\;\;\;\;\;h\left(n\right)=\frac{1}{N}\sum_{k=0}^{N-1}H\left(k\right)e^{jnk2\pi /N},n\in \left[-N+1,N-1\right].\;\;\;\;$$

It should be emphasized that, as [21] proved, the transfer curve of this FIR filter g passes through the frequency setting points H(0),⋯,H(N−1). In other words, its sampled Fourier transform satisfies:

(12) G(jk2π/N)=H(k),k=0,⋯,N−1.

### 4.3. Derivation of the Closed-Form Notch Filter Design

Note that this three-step procedure is a general design for a convolution window based FIR filters. Nevertheless, as a special filter, the notch filter with a controllable notching frequency cannot be directly designed by this procedure. Herein, we propose a measure named *reversed phase-shift combination* to modify this procedure, facilitating that this required notch filter can be efficiently designed in a closed-form way.

Since the transfer curve of the convolution window based filter passes through those frequency points of the vector H, let Δω=2π/N be the frequency unit, if we aim to design a notch filter with the notching frequency located at mΔω (corresponding to the analog frequency $f_{0}=mf_{s}/N$ ), H should be set as:

(13) $$H=[\underset{m}{\underset{︸}{0,\ldots ,0}}\;1\;\;\underset{N-2m-1}{\underset{︸}{0,\ldots ,0}}\;\;\;1\;\underset{m-1}{\underset{︸}{0,\ldots ,0}}].$$

To emphasize, H actually possesses the *point-pass* transfer characteristic rather than the *notch* transfer characteristic. This conversion can be realized by the following *reversed phase-shift combination* measure.

This measure requires that H be detached into two vectors $H_{1},H_{2}$ satisfying $H_{1}\left(k\right)=H_{2}\left(N-k\right)$, that is,

(14) $$\begin{array}{cc} H_{1} & =[\underset{m}{\underset{︸}{0,\ldots ,0}}\;1\;\;\underset{N-m-1}{\underset{︸}{0,\ldots ,0}}\;\;\;];\\ H_{2} & =[\underset{N-m}{\underset{︸}{0,\ldots ,0}}\;1\;\underset{m-1}{\underset{︸}{0,\ldots ,0}}]. \end{array}$$

For example, for the case of the order N=16,m=3, $H_{1}$ and $H_{2}$ should be specified as:

(15) $$\begin{array}{cc} H_{1} & =[0\;0\;0\;1\;\;0\;0\;0\;0\;\;0\;0\;0\;0\;\;0\;0\;0\;0];\\ H_{2} & =[0\;0\;0\;0\;\;0\;0\;0\;0\;\;0\;0\;0\;0\;\;0\;1\;0\;0]. \end{array}$$

Further, one can derive that substituting Equation (14) into Equation (11) and combining with Equation (8) yields two sub-filters $g_{1}^{\prime }$ and $g_{2}^{\prime }$.

(16) $$\begin{array}{cc} \;\;\;\;\;g_{1}^{\prime }\left(n\right) & =\frac{w_{c}\left(n\right)}{N}e^{j2\pi nm/N},n\in \left[-N+1,N-1\right];\\ \;\;\;\;\;g_{2}^{\prime }\left(n\right) & =\frac{w_{c}\left(n\right)}{N}e^{-j2\pi nm/N},n\in \left[-N+1,N-1\right]. \end{array}$$

**Figure 4:**
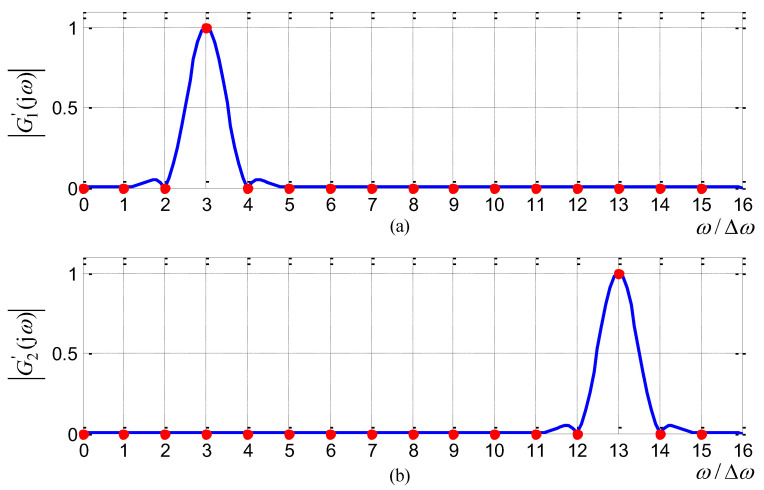
Transfer curves of two sub-filters (N=16, m=3): (**a**) sub-filter $g_{1}^{\prime }$; (**b**) sub-filter $g_{2}^{\prime }$.

For the case of N=16,m=3, these two transfer curves $|G_{1}^{\prime }\left(j\omega \right)|$, $|G_{2}^{\prime }\left(j\omega \right)|$ are plotted in Figure 4.

From Figure 4, one can observe that $|G_{1}^{\prime }\left(j\omega \right)|$, $|G_{2}^{\prime }\left(j\omega \right)|$ (whose filter coefficients follow the closed-formed formula in (16)) strictly pass through the specified frequency points at 3Δω and 13Δω, respectively. Meanwhile, as Figure 4 illustrates, $|G_{1}^{\prime }\left(j\omega \right)|$ and $|G_{2}^{\prime }\left(j\omega \right)|$ are symmetric with ω=π.

Generally speaking, since *m* is an integer in the interval [0,N−1], only *N* notching frequencies are realizable following the aforementioned three-step procedure, which does not meet the 2nd requirement addressed in Section 4.1.

In order to realize that the notching frequency $f_{0}$ can be flexibly moved to any position, we apply a *reversed phase shift operation* on these two sub-filters $g_{1}^{\prime }$ and $g_{2}^{\prime }$ as:

(17) $$\begin{array}{cc} \;\;\;\;\;g_{1}^{\prime \prime }\left(n\right) & =g_{1}^{\prime }\left(n\right)e^{j\lambda \Delta \omega n},n\in \left[-N+1,N-1\right];\\ \;\;\;\;\;g_{2}^{\prime \prime }\left(n\right) & =g_{2}^{\prime }\left(n\right)e^{-j\lambda \Delta \omega n},n\in \left[-N+1,N-1\right]. \end{array}$$

As is well known, in terms of the Fourier transform’s property, the phase shift operation in the time domain gives rise to a shift in the frequency domain. Therefore, the transfer curve $G_{1}^{\prime \prime }\left(j\omega \right)$ is acquired by translating with the λΔω distance from $G_{1}^{\prime }\left(j\omega \right)$ in the right direction, whereas $G_{2}^{\prime \prime }\left(j\omega \right)$ is acquired by translating with λΔω distance from $G_{2}^{\prime }\left(j\omega \right)$ in the left reversed direction.

**Figure 5:**
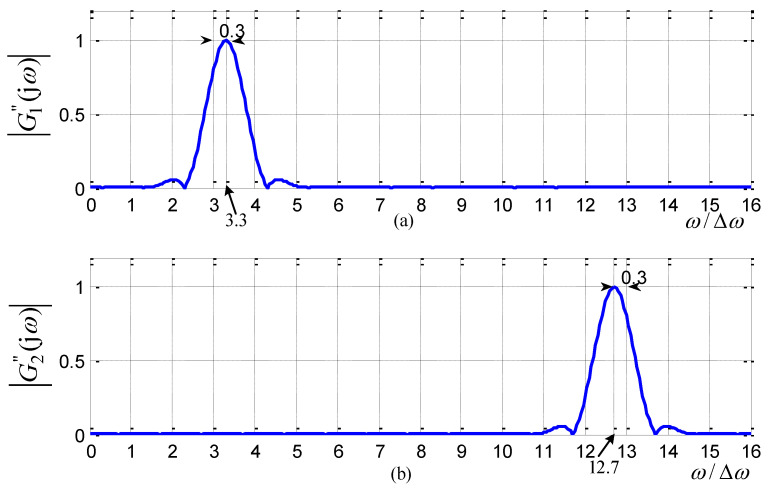
Transfer curves of two sub-filters (N=16, m=3, λ=0.3): (**a**) sub-filter $g_{1}^{\prime \prime }$; (**b**) sub-filter $g_{2}^{\prime \prime }$.

For the case of the order N=16,m=3,λ=0.3, the transfer curves $|G_{1}^{\prime \prime }\left(j\omega \right)|$, $|G_{2}^{\prime \prime }\left(j\omega \right)|$ are plotted in Figure 5.

From Figure 5, it is verified that the notching frequencies $|G_{1}^{\prime \prime }\left(j\omega \right)|$, $|G_{2}^{\prime \prime }\left(j\omega \right)|$ are exactly located at the expected positions 3.3Δω, 12.7Δω.

Summing up these two filters $g_{1}^{\prime \prime }$, $g_{2}^{\prime \prime }$ yields a real-valued filter $g^{\prime \prime }\left(n\right)$ as

(18) $$g^{\prime \prime }\left(n\right)=g_{1}^{\prime \prime }\left(n\right)+g_{2}^{\prime \prime }\left(n\right),n\in \left[-N+1,N-1\right].$$

Further, subtracting $g^{\prime \prime }\left(n\right)$ from the all-pass filter, whose impulse response is δ(n), yields the final real-valued notch filter g, that is,

(19) $$g\left(n\right)=\delta \left(n\right)-g^{\prime \prime }\left(n\right),n\in \left[-N+1,N-1\right].$$

Combining Equations (16)–(18) with (19), one can deduce a closed-form expression of the final notch filter coefficient g(n) as (see Appendix A for detailed deductions):

(20) $$g\left(n\right)=\left\{\begin{array}{c} (N-2)/N,\;n=0;\\ -\frac{2w_{c}\left(n\right)}{N}\cos\left[\frac{2(m+\lambda )n\pi }{N}\right],\mathrm{else}. \end{array}\right.$$

**Figure 6:**
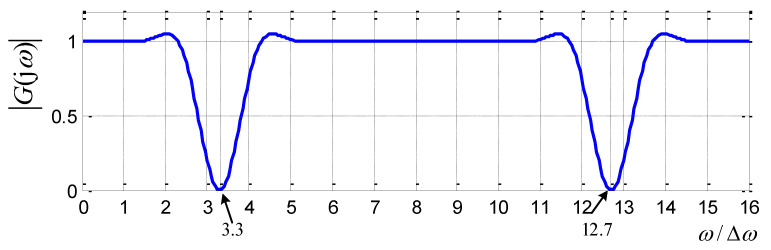
Transfer curve of the notch filter (N=16, m=3, λ=0.3).

Substituting $N=16,w_{c}\left(n\right),m=3,\lambda =0.3$ into Equation (20) and implementing Fourier transform on g(n), one can plot the transfer curve of the final real-valued notch filter, as Figure 6 illustrates.

Moreover, its notching frequencies fall exactly at the expected positions 3.3Δω and 12.7Δω (no more than the mirror frequency of 3.3Δω).

### 4.4. Extra Data Extensions for Linear-Phase Characteristic

Linear-phase characteristic is of vital importance in speech processing systems. A nonlinear-phase system means that individual frequency components experience different delays.

As is known, for an FIR filter, if its coefficients satisfy the symmetry, it possesses linear-phase characteristic. This symmetry can be readily inferred from Equation (20) that

(21) g(n)=g(−n),n=−N+1,…,N−1,

indicating that the proposed filter is of linear-phase characteristic.

To emphasize, the linear-phase characteristic not only depends on the notch filter itself, but is also related to the data feed mode. Since the proposed scheme adopts the segmentation based data feed mode to the filter, this kind of truncation also brings a nonlinear-phase effect. Hence, we adopt the data extension measure to avoid this side effect.

Since this linear-phase notch filter is of the length (2N−1), its group delay τ equals (2N−1−1)/2=N−1 samples. Hence, to ensure the consistency of input data, for the current *i*-th subsegment $x_{i}=\left[x_{i}\left(0\right),x_{i}\left(1\right),\ldots ,x_{i}\left(P-1\right)\right]=\left[x\left(iP\right),x\left(iP+1\right),\ldots ,x\left(iP+P-1\right)\right]$, it not only needs to link backward to the last (N−1) samples of the (i−1)-th subsegment, but also needs to link forward to the first (N−1) samples of the (i+1)-th subsegment. In other words, the extended segment of $x_{i}$ is actually a (P+2N−2)-length sequence expressed as $x_{i}^{\prime }=\left[x_{i}^{b}\;x_{i}\;x_{i}^{f}\right]$, which is structured as (P≫N):

(22) $$\begin{array}{cc} x_{i}^{\prime }= & \left[x_{i}^{b}\;\;\;x_{i}\;\;\;x_{i}^{f}\right]\\ = & [\underset{N-1\;\mathrm{extended}\;\mathrm{samples}}{\underset{︸}{x_{i-1}\left(P-N+1\right),\ldots ,x_{i-1}\left(P-1\right)}},\underset{P\;\mathrm{samples}}{\underset{︸}{x_{i}\left(0\right),x_{i}\left(1\right),\ldots ,x_{i}\left(P-1\right)}},\\ \; & \underset{N-1\;\mathrm{extended}\;\mathrm{samples}}{\underset{︸}{x_{i+1}\left(0\right),\ldots ,x_{i+1}\left(N-2\right)}}]. \end{array}$$

Further, the extensions of two extra (N−1)-length sequences $x_{i}^{b}=\left[x_{i-1}\left(P-N+1\right),\ldots ,x_{i-1}\left(P-1\right)\right]$ and $x_{i}^{f}=\left[x_{i+1}\left(0\right),\ldots ,x_{i+1}\left(N-2\right)\right]$, are also based on the consideration of removing the transient process of FIR filtering. Specifically, as is well known, an FIR filter’s output equals the convolution between its impulse response and the input sequence. Hence, since our proposed filter includes (2N−1) tap coefficients and the input extended sequence $x_{i}^{\prime }$ is of the length (P+2N−2), its output sequence (also the convolution result of these two parts) is of the length (P+2N−2)+(2N−1)−1=P+4N−4, which is structured as:

(23) $$\begin{array}{cc} y_{i}^{\prime } & =[y_{i}^{t_{1}}\;\;\;y_{i}\;\;\;y_{i}^{t_{2}}]\\ \; & =[\underset{2N-2\;\mathrm{transient}\;\mathrm{samples}}{\underset{︸}{y_{i}^{t_{1}}\left(0\right),\ldots ,y_{i}^{t_{1}}\left(2N-3\right)}},\underset{P\;\mathrm{remained}\;\mathrm{samples}}{\underset{︸}{y_{i}\left(0\right),y_{i}\left(1\right),\ldots ,y_{i}\left(P-1\right)}},\underset{2N-2\;\mathrm{transient}\;\mathrm{samples}}{\underset{︸}{y_{i}^{t_{2}}\left(0\right),\ldots ,y_{i}^{t_{2}}\left(2N-3\right)}}]. \end{array}$$

Moreover, a (2N−1)-length FIR filter contains (2N−2) digital delay elements. In other words, this filter has to experience a transient process with the duration of (2N−2) samples (i.e.,$y_{i}^{t_{2}}$ or $y_{i}^{t_{2}}$ in Equation (23)), whether at the beginning stage or at the ending stage. Therefore, if we take the middle *P* samples $y_{i}\left(0\right),\ldots ,y_{i}\left(P-1\right)$ among $y_{i}^{\prime }$ as the current output, both the first (2N−2) transient output samples and the last (2N−2) transient output samples will exactly be discarded, indicating that the remained *P* samples are surely steady-state outputs. Consequently, the entire spliced waveform, which links all these *P*-length steady-state output sequences, will not exhibit obvious truncation effect. In this way, the linear-phase characteristic of the proposed scheme is guaranteed.

### 4.5. Analysis of the Proposed Notch Filter

Let us go back to Section 4.1 to verify whether the proposed notch filter satisfies these requirements or not.

As to the 1st requirement, since all the tap coefficients are calculated in terms of the closed-form formula Equation (20), this design is of very low complexity.

As to the 2nd requirement, since the *phase-shift combination* technique is proposed, which ensures that the notching frequency can be specified to arbitrary position just by setting the integer *m*, and the fractional offset λ.

As to the 3rd requirement, since [21] proved that the convolution window based filter’s transfer curve passes through the frequency setting points, which ensures that the filter’s attenuation value can reach −330 dB in combination with the *reversed phase-shift combination* technique.

As to the 4th requirement, the proposed notch filter’s coefficient symmetry, the segmenting and splicing operations and the extra data extension together ensure that the resulting speech does not include nonlinear-phase distortion.

Due to the above merits, the proposed segmented notch filter based howling suppression scheme possesses the following superiorities over the traditional adaptive filter based scheme:

1. The proposed scheme exhibits stronger robustness to the time-varying characteristic of the echo channel H(z) (arising from its unpredictability) than the adaptive filter based scheme. When howling happens, both the howling frequency and the time-variant impulse response of the echo channel are essentially the acute reflection of the feedback channel. Meanwhile, the proposed scheme employs a spectrum corrector to estimate the howling frequency, whereas the traditional scheme employs the adaptive filter to track the echo’s impulse response. The former is implemented in the frequency domain, whereas the latter is implemented in the time domain. Hence, the former is more robust.
2. The proposed scheme has a better real-time performance than the adaptive filter based scheme. Since our scheme actually only consumes low complexity, all the involved operations are simple (i.e., segmentation and splicing, spectrum correction, closed-form notch filter design and FIR filtering), whereas the latter consumes high complexity for the sake of balancing the trade-off between the parameter settings of step size and the steady-state residual error.
3. The proposed scheme can guarantee the linear-phase characteristic, whereas the adaptive filter based scheme cannot. This is because the former adopts a linear-phase closed-form FIR filter design and proper data extension, whereas the latter adopts IIR notch design, which inevitably creates linear-phase distortion.

This section may be divided by subheadings. It should provide a concise and precise description of the experimental results and their interpretation, as well as the experimental conclusions that can be drawn.

## 5. Numerical Results

### 5.1. Descriptions of Howling Suppression Generation

Denote s(n), x(n), y(n) as the original speech, the howling mixed speech and the output speech recovered by the proposed scheme, respectively. Specify the sampling rate as $f_{s}$ = 16 kHz. We have a male speech s(n) lasting for T=7.625 s, which utters several Chinese words (thus it covers $L=f_{s}\times T$ = 122,000 samples). For our proposed scheme, the subsegment length is set as P=2000, the filter order is set as N=32 (thus P≫N) and the *N*-length window f involved in the convolution window Formula (9) is set as the hamming window. For the sake of simplicity, the forward gain is dealt as G(z)=1.

On the basis of the above parameterizations, we added the howling component with the frequency $f_{0}=1590$ Hz, amplitude A=1 at the time instant t=1.5 s (i.e., the $L_{h}$ = 24,000-th sample, corresponding to the (24,000/P + 1) = 13-th subsegment) to s(n). The recovery signal-to-noise ratio (RSNR) is utilized to evaluate the proposed scheme’s performance, which is defined as:

(24) $$RSNR=10lg\frac{\sum_{n=L_{h}}^{L}s^{2}\left(n\right)}{\sum_{n=L_{h}}^{L}{\left[y\left(n\right)-s\left(n\right)\right]}^{2}}.$$

Besides, we will also investigate the transfer characteristic of the closed-form notch filter, the RSNR results versus individual howling frequencies, individual filter orders *N* and individual data extension lengths. Moreover, the recovered waveforms near subsegment edges will be utilized to reflect the phase-distortion degree.

To emphasize, as mentioned above, both the howling frequency and the time-variant impulse response of the echo channel are essentially the acute reflection of the feedback channel. Hence, the existing howling suppression methods [4,17,27,28] placed more emphasis on the system identification result of the echo channel rather than the waveform recovery quality. Moreover, the notch filter employed in the existing adaptive filter-based schemes [16,29,30] is mainly utilized to compensate the system identification deviation of the echo channel, whereas our proposed scheme’s notch filter is directly utilized to suppress the howling component arising from the echo channel itself. Since the prerequisites and dependent conditions of the these algorithms are distinct, it is not practical to conduct comparisons among them.

### 5.2. Comparison between the Proposed Notch Filter and the Conventional IIR Notch Filter

As a reference, the second-order IIR model was employed to design an IIR notch filter, which is formulated as [31,32]:

(25) $$G\left(z\right)=\frac{\left(1-r_{z}e^{j\omega_{c}}z^{-1}\right)\left(1-r_{z}e^{-j\omega_{c}}z^{-1}\right)}{\left(1-r_{p}e^{j\omega_{c}}z^{-1}\right)\left(1-r_{p}e^{-j\omega_{c}}z^{-1}\right)}.$$

**Figure 7:**
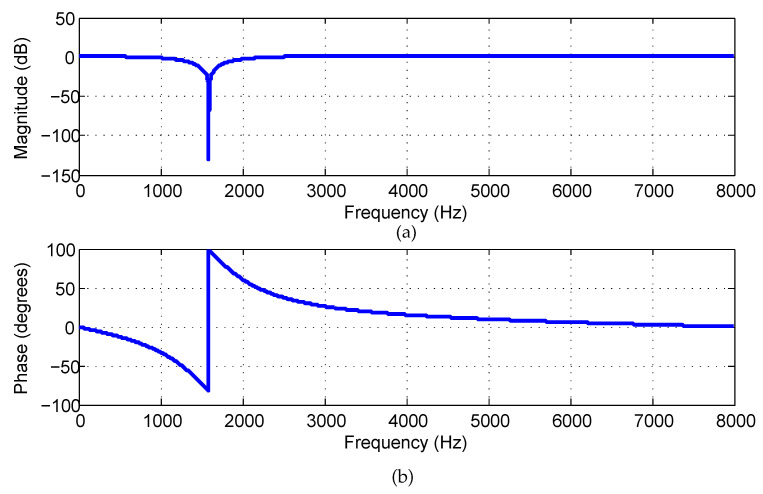
Conventional IIR notch filter frequency response: (**a**) The attenuation curve; (**b**) The phase-curve.

Specifically, substituting $r_{z}=1,r_{p}=0.8,\omega_{c}=2\pi f_{0}/f_{s}=0.1988\pi$ into Equation (25) yields the IIR notch filter, whose attenuation curve and phase response curve are plotted in Figure 7a,b, respectively.

**Figure 8:**
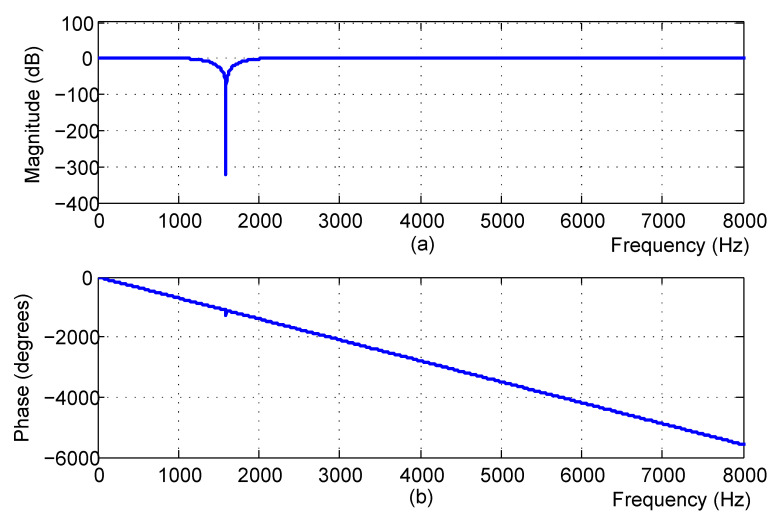
The proposed notch filter frequency response: (**a**) The attenuation curve; (**b**) The phase-curve.

In contrast, following the proposed procedure in Section 3.2, we can acquire m=3, λ=0.18 by means of the spectrum corrector. Further, substituting $N,m,\lambda ,w_{c}\left(n\right)$ into Equation (20) yields the final notch filter g(n), whose attenuation curve and phase response curve are plotted in Figure 8a,b, respectively.

**Figure 9:**
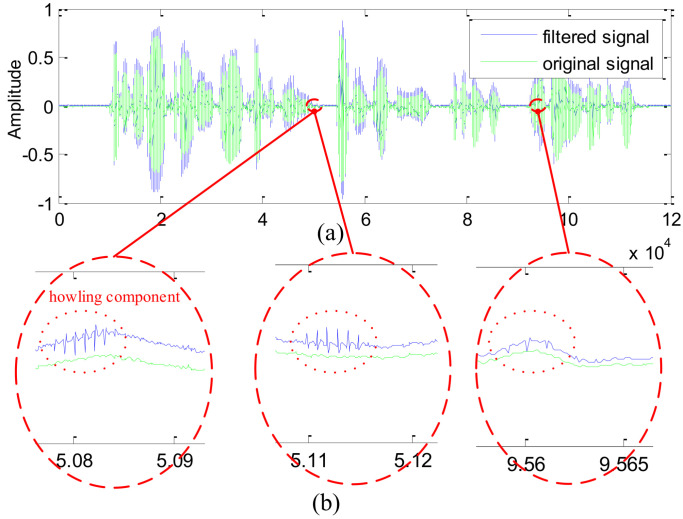
The signal waveforms filtered by the IIR notch filter: (**a**) The original waveform and the filtered waveform; (**b**)The enlarged waveform.

**Figure 10:**
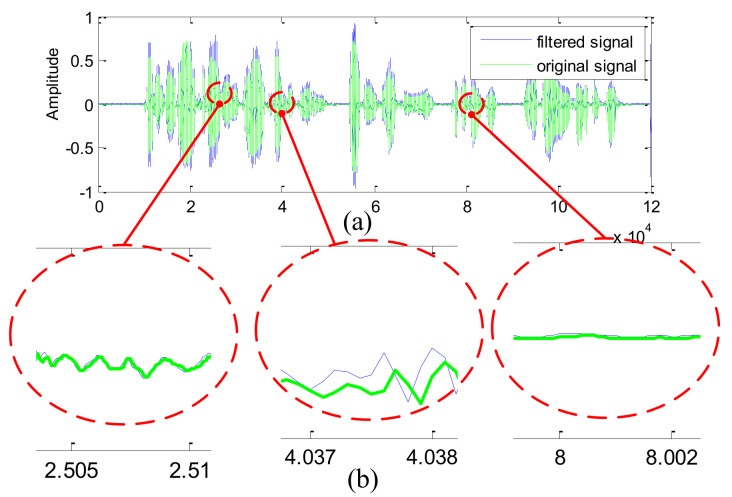
The signal waveforms filtered by the proposed notch filter: (**a**) The original waveform and the filtered waveform; (**b**)The enlarged waveform.

Further, we also present the comparison between the waveform recovered by the IIR notch filter (see Figure 9) and the waveform recovered by the proposed notch filter (see Figure 10).

From Figure 7 and Figure 8, the following conclusions can be drawn:

1. The transitional band of the IIR notch filter is slightly wider than that of the proposed filter, which inevitably damages more useful frequency components near the notch position.
2. Focusing on the notching position, the attenuation value of the IIR filter is about −150 dB, whereas the attenuation value of the proposed filter is lower than −330 dB. As a result, the howling component still appears on the former recovered waveform (see the enlarged waveform in Figure 9b), whereas no howling component is visible on the latter recovered waveform (see the enlarged waveform in Figure 10b).

### 5.3. Demonstration of the Proposed Closed-Form Notch Filter Based Scheme

**Figure 11:**
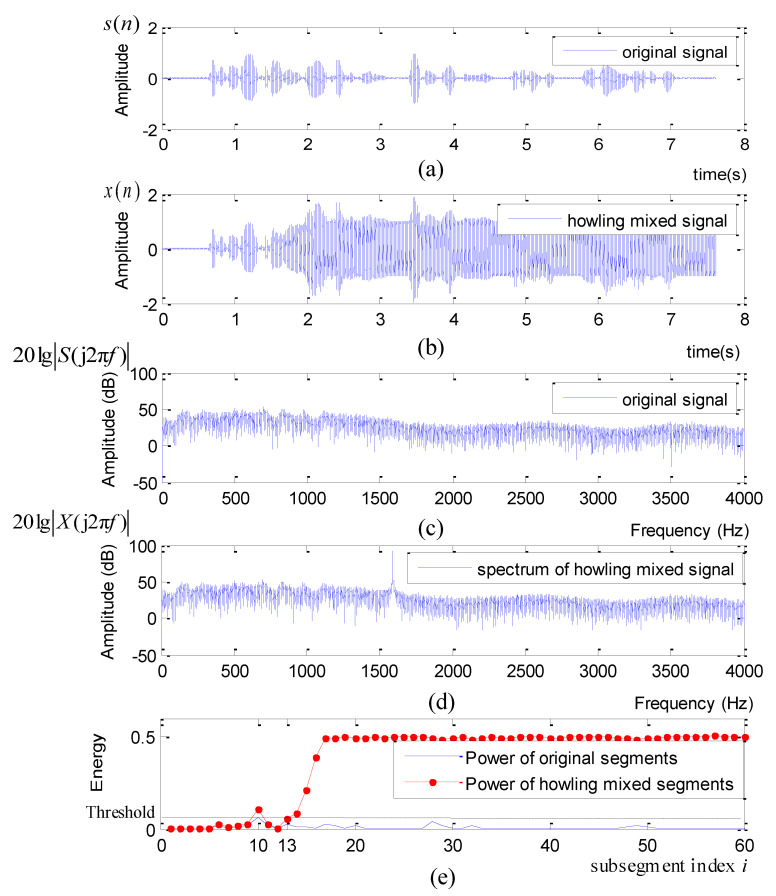
Energy Detector: (**a**) The original signal waveform; (**b**) The howling mixed signal waveform; (**c**) The spectrum of the original signal; (**d**) The spectrum of the howling mixed signal; (**e**) The energy distribution of the original signal and the howling mixed signal.

Following the procedure addressed in Section 3 and Figure 3, we present the original waveform s(n), the howling mixed waveform x(n), the spectrum of s(n), the spectrum of x(n) and the power distribution calculated by the energy detector in Figure 11a–e, respectively.

Comparing Figure 11c with Figure 11d, one can notice that the spectral peak appears at $f_{0}=1590$ Hz in the log spectrum 20lg|X(j2πf)|. Moreover, as Figure 11 plots, the energy detector correctly judged that the howling occurs at the 13-th subsegment.

### 5.4. Comparison of Different Howling Frequencies

To investigate the proposed scheme’s recovery quality versus different howling frequencies, we altered a series of howling frequencies ranging from 1290 Hz to 3390 Hz, which were listed in Table 1 together with their RSNR results.

It can be discovered from Table 1 that these RSNRs only fluctuate within a narrow range around 22 dB, indicating that the proposed notch filter is insensitive to the howling frequency. Further, since the closed-form Equation (20) is explicit, this robustness also reflects that the spectrum corrector works well for any howling frequencies.

### 5.5. Comparison of Different Filter Orders

To investigate the proposed scheme’s recovery quality versus the filter orders *N*, we altered a series of the filter orders *N* from 32 to 1024, which are listed in Table 2 together with their RSNR results.

It can be discovered from Table 2 that these RSNRs fluctuate within a narrow range around 22 dB, indicating that the proposed notch filter is insensitive to the filter order.

This robustness lies in the following two reasons. On one side, as the filter order *N* increases, the transition band surely gets narrower, which helps to improve the RSNR; On the other side, due to the adoption of the data segmentation technique, this increasing filter order also widens the FIR notch filter’s transient process, which tends to degrade the recovery quality. Due to this compensation effect, the RSNRs almost remains unchanged. Hence, practically speaking, the lowest order *N* is preferred, since this selection means the lowest system cost.

### 5.6. Comparison of Different Data Extension Lengths

To investigate the proposed scheme’s recovery quality versus the data extension lengths, we altered a series of the data extension lengths from N−1 to 4N−1. Specifically, independent of the data extension length, the middle P=2000 samples of the convolution results are consistently taken as the current subsegment’s output.

On this basis, the RSNR results are listed in Table 3, from which one can notice that these RSNRs almost remained unchanged with the data extension lengths. This robustness arises from the fact, as Section 4.4 analyzes, that the minimum data extension length (N−1) actually fully takes consideration of the FIR notch filter’s (2N−2) transient samples whether at the beginning stage or at the ending stage.

### 5.7. Demonstration of Linear-Phase Recovery Effect

**Figure 12:**
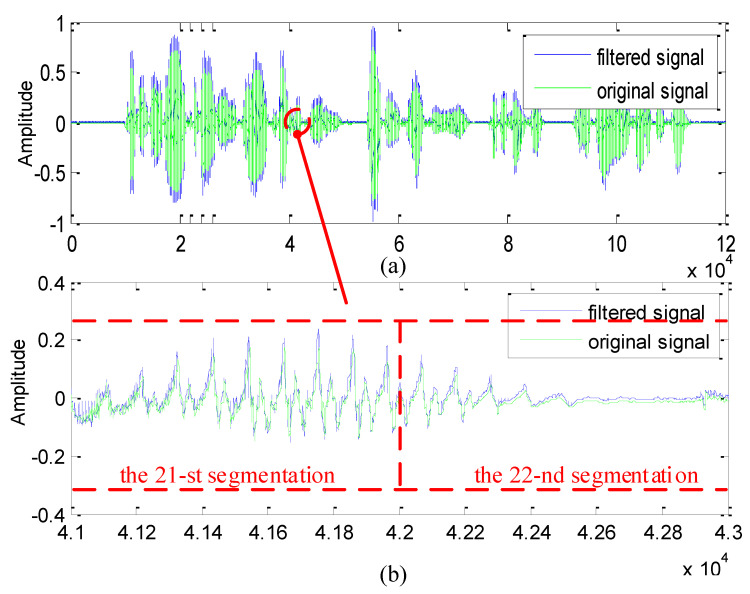
The original waveform and the recovered waveform. (**a**) The entire waveforms. (**b**) Edges linking to the 21-st subsegment and the 22-nd subsegment.

Note that, the data feeding off howling detection is based on the subsegment-by-subsegment mode, which indicates that step-like change inevitably occurs at those positions linking two adjacent subsegments if the filter does not possess the linear-phase characteristics. Hence, to verify the proposed scheme’s linear-phase characteristic, we focus on whether acute jumps appear at some edges, linking to adjacent subsegments of the the recovered waveform, as illustrated in Figure 12.

As Figure 12 depicts, it is clear that an almost acute jump does not appear at the edge linking the 21-st subsegment to the 22-nd subsegment. Actually, we also investigated all the edges and no acute jumps were found, which confirms the proposed scheme’s linear-phase characteristic.

## 6. Conclusions

This paper proposes a scheme of howling detection and howling suppression applied in hearing aids, which incorporates the core closed-form FIR notch filter design with the spectrum correction, segmentation and splicing techniques.

This scheme not only essentially solves the existing echo cancellation based scheme’s problems of insufficient howling suppression, phase distortion and so forth, but also consumes a low complexity due to the fact that the core notch filter can be designed by a simple analytic formula. We have conducted a series of numerical simulations in verifying these merits, which helps technique innovation for hearing aids. Practically, the proposed algorithm can be implanted in a system-on-chip (SOC), which possess high flexibility of functionality updating, and low power consumption. As the core component, the proposed howling detection possesses vast potential for the practical applications of future digital hearing aids.

## Figures and Tables

**Table 1:** Howling frequencies $f_{0}$ and RSNR (N=32).

$f_{0}$ (Hz)	RSNR (dB)
1290	21.28
1590	21.92
1890	21.97
2190	22.11
2490	22.18
2790	22.12
3090	22.04
3390	22.01

**Table 2:** Filter order *N* and RSNR ($f_{0}=1590$ Hz).

Order *N*	RSNR (dB)
32	21.92
64	21.83
128	21.75
256	22.43
512	22.81
1024	20.97

**Table 3:** Data extension length and RSNR($f_{0}=1590$ Hz, N=32).

Length	RSNR (dB)
31	21.92
48	21.92
63	21.92
96	21.92
127	21.92

## Data Availability

Not applicable.

## Appendix A

Given the normalized convolution window $w_{c}\left(n\right)$, the frequency sampling length *N*, the frequency index m∈{0,…,N−1}, the frequency offset λ∈[−0.5,0.5), then, the coefficient of the final convolution window based FIR notch filter equals

(A1) $$g\left(n\right)=\left\{\begin{array}{c} (N-2)/N,\;n=0\\ -\frac{2w_{c}\left(n\right)}{N}cos\left[\frac{2(m+\lambda )n\pi }{N}\right],\mathrm{else} \end{array}\right..$$

Combining Equations (15) and (17), we have

(A2) $$\begin{array}{cc} \;\;\;\;g_{1}^{\prime \prime }\left(n\right) & =w_{c}\left(n\right)e^{j(m+\lambda )\Delta \omega n},n\in \left[-N+1,N-1\right];\\ \;\;\;\;g_{2}^{\prime \prime }\left(n\right) & =w_{c}\left(n\right)e^{j(N-m-\lambda )\Delta \omega n},n\in \left[-N+1,N-1\right]. \end{array}$$

Substituting Δω=2π/N and Equation (A2) into Equation (18) yields

(A3) $$g^{\prime \prime }\left(n\right)=2\frac{w_{c}\left(n\right)}{N}\cos\left[\frac{2\pi (m+\lambda )n}{N}\right],n\in \left[-N+1,N-1\right].$$

Since the convolution window $w_{c}$ is normalized by $C=\sum_{n=0}^{N-1}f(n)$, its center element $w_{c}\left(0\right)=1$. Finally, substituting $w_{c}\left(0\right)=1$ and Equation (A3) into Equation (19) yields

(A4) $$g\left(n\right)=\left\{\begin{array}{c} (N-2)/N,\;n=0\\ -\frac{2w_{c}\left(n\right)}{N}\cos\left[\frac{2(m+\lambda )n\pi }{N}\right],\mathrm{else} \end{array}\right..$$

## References

[1] Dhawan R., Mahalakshmi P. Digital filtering in hearing aid system for the hearing impaired. Proceedings of the 2016 International Conference on Electrical, Electronics, and Optimization Techniques (ICEEOT).

[2] Mullins K.A. Design of a digital hearing aid. Proceedings of the IEEE Technical Applications Conference (Northcon/96), Conference Record.

[3] Luo H., Arndt H. Digital signal processing technology and applications in hearing aids. Proceedings of the 6th International Conference on Signal Processing.

[4] Shen H., Zhang L. A new variable step-size algorithm onacoustic feedback suppression for digital hearing aids. Proceedings of the 2014 International Conference on Audio, Language and Image Processing.

[5] Crummer R.W., Hassan G.A. (2004). Diagnostic approach to tinnitus. Am. Fam. Physician.

[6] Chung K. (2004). Challenges and recent developments in hearing aids: Part ii. feedback and occlusion effect reduction strategies, laser shell manufacturing processes, and other signal processing technologies. Trends Amplif..

[7] Ngo K., Waterschoot T.V., Christensen M.G., Moonen M., Jensen S.H. (2013). Improved prediction error filters for adaptive feedback cancellation in hearing aids. Signal Process..

[8] Ma G., Gran F., Jacobsen F., Agerkvist F.T. (2011). Adaptive feedback cancellation with band-limited LPC vocoder in digital hearing aids. IEEE Trans. Audio Speech Lang. Process..

[9] Guo M., Jensen S.H., Jensen J. (2012). Novel acoustic feedback cancellation approaches in hearing aid applications using probe noise and probe noise enhancement. IEEE Trans. Audio Speech Lang. Process..

[10] Strasser F., Puder H. (2015). Adaptive feedback cancellation for realistic hearing aid applications. IEEE/ACM Trans. Audio Speech Lang..

[11] Friedlander B., Smith J. (1984). Analysis and performance evaluation of an adaptive notch filter. IEEE Trans. Inf. Theory.

[12] Borio D. (2016). Loop analysis of adaptive notch filters. IET Signal Process..

[13] Waterschoot T., Moonen M. (2011). Fifty years of acoustic feedback control: State of the art and future challenges. Proc. IEEE.

[14] Hamidia M., Amrouche A. (2016). Improved variable step-size nlms adaptive filtering algorithm for acoustic echo cancellation. Digit. Signal Process..

[15] Liang R., Wang X., Wang Q., Zou C. (2017). A joint echo cancellation algorithm for quick suppression of howls in hearing aids. IEEJ Trans. Electr. Electron. Eng..

[16] Sogami A., Kawamura A., Iiguni Y. (2011). A high speech quality distance-based howling canceller with adaptive cascade notch filter and silent pilot signal. ICE Trans. Fundam. Electron. Commun. Comput. Sci..

[17] Pandey A., Mathews V.J. Howling suppression in hearing aids using least-squares estimation and perceptually motivated gain control. Proceedings of the 2006 IEEE International Conference on Acoustics Speech and Signal Processing Proceedings.

[18] Mcclellan J., Parks T. (1973). A united approach to the design of optimum FIR linear-phase digital filters. IEEE Trans. Circuit Theory.

[19] Zahradnik P., Vlcek M. (2004). Fast analytical design algorithms for FIR notch filters. IEEE Trans. Circuits Syst. Regul. Pap..

[20] Zahradnik P., Vlcek M. (2006). An analytical procedure for critical frequency tuning of fir filters. IEEE Trans. Circuits Syst. II Express Briefs.

[21] Huang X., Jing S., Wang Z., Xu Y., Zheng Y. (2016). Closed-form fir filter design based on convolution window spectrum interpolation. IEEE Trans. Signal Process..

[22] Bustamante D.K., Worrall T.L., Williamson M.J. Measurement and adaptive suppression of acoustic feedback in hearing aids. Proceedings of the 1989 International Conference on Acoustics, Speech, and Signal Processing (ICASSP-89).

[23] Zhang F., Geng Z., Yuan W. (2001). The algorithm of interpolating windowed fft for harmonic analysis of electric power system. IEEE Trans. Power Deliv..

[24] Huang X., Xu J., Liu Y. (2019). Underdetermined speech blind identification based on spectrum correction and phase coherence criterion. IEEE Access.

[25] Haab L., Lehser C., Bernarding C., Corona-Strauss F.I., Seidler H., Hannemann R., Straussa D.J. (2019). Implementation and long-term evaluation of a hearing aid supported tinnitus treatment using notched environmental sounds. IEEE J. Transl. Eng. Health Med..

[26] Tsuchiya M., Sasaoka N., Itoh Y., Shiogai K., Kobayashi M. Bias free adaptive notch filter based on sscf algorithm with decorrelation parameter. Proceedings of the 2015 15th International Symposium on Communications and Information Technologies (ISCIT).

[27] Spriet A., Eneman K., Moonen M., Wouters J. Objective measures for real-time evaluation of adaptive feedback cancellation algorithms in hearing aids. Proceedings of the 2008 16th European Signal Processing Conference.

[28] Kar A., Ankitab A., Swamy M.N.S. (2020). Automatic tap-length adjustment of adaptive filter for feedback attenuation in hearing aids. Appl. Acoust..

[29] Loetwassana W., Punchalard R., Lorsawatsiri A., Koseeyaporn J., Wardkein P. Adaptive howling suppressor in an audio amplifier system. Proceedings of the 2007 Asia-Pacific Conference on Communications.

[30] Feng Y.Q., Tang G.C., Liang R.Y., Wang Q.Y. An improved echo cancellation algorithm for hearing aids. Proceedings of the 2015 International Conference on Electronics, Electrical Engineering and Information Science (EEEIS2015).

[31] Carney R. (1963). Design of a digital notch filter with tracking requirements. IEEE Trans. Space Electron. Telem..

[32] Leotwassana W., Punchalard R., Silaphan W. Adaptive howling canceller using adaptive iir notch filter: Simulation and implementation. Proceedings of the International Conference on Neural Networks & Signal Processing.

